# Fractal and multifractal analysis of PET/CT images of metastatic melanoma before and after treatment with ipilimumab

**DOI:** 10.1186/s13550-016-0216-5

**Published:** 2016-07-29

**Authors:** Christina-Marina Breki, Antonia Dimitrakopoulou-Strauss, Jessica Hassel, Theoharis Theoharis, Christos Sachpekidis, Leyun Pan, Astero Provata

**Affiliations:** 1 Institute of Nanoscience and Nanotechnology, National Center for Scientific Research “Demokritos”, Athens, Greece; 2 Department of Informatics & Telecommunications, National and Kapodistrian University of Athens, Athens, Greece; 3 Clinical Cooperation Unit Nuclear Medicine, German Cancer Research Center (DKFZ), DE-69120 Heidelberg, Germany; 4 National Center for Tumour Disease, Heidelberg, Germany and Department of Dermatology, University Hospital Heidelberg, Heidelberg, Germany; 5 Visual Computing Laboratory, Department of Computer and Information Science, Norwegian University of Science and Technology, Trondheim, Norway; 6 Department of Nuclear Medicine, Inselspital, University Hospital and University of Bern, Bern, Switzerland

**Keywords:** PET/CT imaging, Metastatic melanoma, Ipilimumab, Fractal dimensions, Multifractal spectrum, Kinetic Monte Carlo simulations

## Abstract

**Background:**

PET/CT with F-18-fluorodeoxyglucose (FDG) images of patients suffering from metastatic melanoma have been analysed using fractal and multifractal analysis to assess the impact of monoclonal antibody ipilimumab treatment with respect to therapy outcome.

**Results:**

Thirty-one cases of patients suffering from metastatic melanoma have been scanned before and after two and after four cycles of treatment. For each patient, we calculated the fractal and multifractal dimensions using the box-counting method on the digitalised PET/CT images of all three studies to assess the therapeutic outcome. We modelled the spreading of malignant cells in the body via kinetic Monte Carlo simulations to address the dynamical evolution of the metastatic process and to predict the spatial distribution of malignant lesions. Our analysis shows that the fractal dimensions which describe the tracer dispersion in the body decrease consistently with the deterioration of the patient’s therapeutic outcome condition. In 20 out of 24 cases, the fractal analysis results match those of the treatment outcome as defined by the oncologists, while 7 cases are considered as special cases because the patients had non-tumour-related findings or side effects which affect the results. The decrease in the fractal dimensions with the deterioration of the patient conditions (in terms of disease progression) is attributed to the hierarchical localisation of the tracer which accumulates in the affected lesions and does not spread homogeneously throughout the body. Fractality emerges as a result of the migration patterns which the malignant cells follow for propagating within the body (circulatory system, lymphatic system). Analysis of the multifractal spectrum complements and supports the results of the fractal analysis. In the kinetic Monte Carlo modelling of the metastatic process, a small number of malignant cells diffuse through a fractal medium representing the blood circulatory network. Along their way, the malignant cells engender random metastases (colonies) with a small probability and, as a result, fractal spatial distributions of the metastases are formed similar to the ones observed in the PET/CT images.

**Conclusions:**

The Monte Carlo-generated spatial distribution of metastases changes with time approaching values close to the ones recorded in the metastatic patients. Thus, we propose that fractal and multifractal analyses have potential applications in quantification of the evaluation of PET/CT images to monitor the disease evolution as well as the response to different medical treatments. The proposed approach, being operator independent, can offer new diagnostic tools in parallel to the visual location of the lesions and may improve multiparameter assessment of FDG PET/CT studies.

## Background

Many constituent networks of the human body present complicated geometric structures which derive from their functional needs, i.e. the geometry of the network serves its functionality. Common examples are (a) the circulatory system which is responsible for the efficient distribution of blood in the body, (b) the respiratory system which coordinates the transport of oxygen in the body and the collection and release of carbon dioxide and (c) the nervous system which controls the dynamic transmission of information via electrical signals in the brain and throughout the body [1]. These three major systems are complex structures with branching and sub-branching down in many hierarchical orders. Branching is considered as a very efficient and energy-saving architecture to transfer matter (or information) from a central source (organ) to a large number of destinations. Because of branching, the body systems are self-similar in many orders of magnitude and exhibit fractal scaling.

In particular, the blood circulatory system distributes the blood in the whole body starting from an organ of large size and reaching individual cells. To achieve this, the distribution network starts from a large artery leaving the heart and branches out in many levels. At each level, the number of branches increases while their diameter decreases in order to reach and feed all cells. This is a mathematical property of fractals in 3D which are constructed as space filling objects [2].

There have been a large number of attempts to quantify the value of the fractal dimensions of the cardiovascular system. Already in 1977, Mandelbrot predicted that the fractal dimension of an arterial tree should be smaller than 3 and he coined the value 2.7 as plausible in refs [2, 3]. Huang et al. measured experimentally a value *d*_f_ = 2.71 for pulmonary arteries and *d*_f_ = 2.69 for pulmonary veins [4]. Helmberger et al. reported fractal dimensions of the lung vessels in pulmonary hypertension patients as *d*_f_ = 2.34, with *d*_f_ increasing to the value 2.37 for patients without pulmonary hypertension [5]. Gil-García et al. showed experimentally that the arterial pattern of the dog kidneys has fractal dimension ~2.7 [6].

The blood circulatory system often serves as a means of transportation for malignant cells to migrate through the body. As stem cancer cells migrate, they colonize distant areas producing metastases, which are the most frequent causes of death for patients with cancer. The molecular mechanisms of metastasis are still not well understood due to their biological complexity, but the migration through the blood circulatory system seems plausible since all body cells come in direct contact and exchange with blood [7]. With this scenario the pattern of migration and colonization of malignant cells should be directly linked with the structure of the distributing blood network. It is then reasonable to search for fractality in the distribution of metastases in the human body, since the transportation network distributing the cancer cells is fractal. In many cases, the evolution of cancer (melanoma in this case) can take a long time and it is a dynamical process. The final distribution is achieved only at the latest stages, while fractality changes during the various stages of the disease, as the spatial distribution of metastatic structures expands in the body.

One molecular imaging technique for the detection of primary tumours and metastases is positron emission tomography (PET) using the radioactive biomarker F-18-fluorodeoxyglucose (FDG). This technique allows the detection of viable tumour tissue and is used for staging and therapy monitoring of melanoma patients since several years [8–13]. In particular, the use of hybrid systems, like PET/CT and PET/MRI, allows a better anatomic allocation of the PET findings and leads to an improvement of both diagnosis and treatment planning [14].

Metastatic melanoma had a poor diagnosis with a median overall survival of less than 1 year, until recently [15]. The conventional treatment consisted in chemotherapy, radiotherapy, high-dose interleukin-2 and best supportive care. An improvement of the overall survival was achieved after the discovery of ipilimumab [15, 16]. Ipilimumab is a fully human, recombinant monoclonal antibody that activates the immune system by targeting cytotoxic T-lymphocyte-associated antigen 4 (CTLA-4). CTLA-4 is a type 1 transmembrane glycoprotein whose expression is primarily restricted to T cells [17]. It is a key negative regulator of T lymphocyte activation via antagonism of CD28-mediated co-stimulation. Melanoma, and tumours in general, can take advantage of this inhibitory mechanism used by the immune system to reduce T cell response. Therefore, the use of blocking antibodies against this inhibitory checkpoint, like ipilimumab, can enhance anti-tumour response.

Bearing these in mind, it is reasonable to use fractal and multifractal analysis as a quantification procedure for the evaluation of FDG images to monitor the disease evolution as well as the patient’s response to different medical treatments [2, 18, 19]. In the current study, we demonstrate the use of fractal/multifractal analysis in detecting the response of 31 melanoma patients to treatment with ipilimumab monoclonal antibody, as will be explained in the sequel. The proposed analysis has the advantage of being operator independent and it offers new diagnostic directions, in parallel to the more laborious approaches of visually locating or manually contouring lesions in images [8].

In the next section, we describe the properties of the patients group and the fractal and multifractal methods used in the analysis of the spatial distribution of the metastatic lesions. We also present the Kinetic Monte Carlo (KMC) method simulating the metastatic evolution. In the “Results” section, we present the main results of our study before and after treatment with the monoclonal antibody. Except from the results of the core group of patients, we also present some specific cases which have non-tumour-related findings. Also we present the results from the Kinetic Monte Carlo model which involves propagation of malignant cells through a fractal network causing occasional metastases and we compare the numerical results to those of the analysis of the PET/CT images. Finally, we recapitulate our main results and discuss open problems.

## Methods

### Patients

The study involved 31 patients (P1, P2, …, P31) who suffered from metastatic melanoma at various stages of the disease. Each patient was scanned once before treatment (study I, baseline scan) and in follow-up, during and after treatment with the monoclonal antibody ipilimumab. Follow-up scans were performed after two cycles of ipilimumab treatment (study II, first follow-up scan), and at the end of the four-cycle treatment (study III, second follow-up scan). The time interval between the first and the second scans was 5 weeks and the first and the third scans 12 weeks after onset to therapy. Due to the tendency of melanoma to produce metastases throughout the body, whole-body scans were performed in all three studies of each patient.

Patients gave written informed consent to participate in the study and to have their medical records released. The study was approved by the Ethical Committee of the University of Heidelberg and the Federal Agency of Radiation Protection.

### Data acquisition

Whole-body PET/CT studies were performed from the skull to the toes with an image duration of 2 min per bed position for the emission scans. A dedicated PET/CT system (Biograph mCT, S128, Siemens Co., Erlangen, Germany) with an axial field of view of 21.6 cm with TruePoint and TrueV, operated in a three-dimensional mode was used. Spatial resolution is 4 mm and the peak noise equivalent count rate (NECR) is 186 kcps at 30.1 kBq/mL [20]. A low-dose attenuation CT (120 kV, 30 mA) was used for attenuation correction of the PET data and for image fusion. All PET images were attenuation-corrected and an image matrix of 400 × 400 pixels was used for iterative image reconstruction. Iterative image reconstruction was based on the ordered subset expectation maximization (OSEM) algorithm with six iterations and 12 subsets. As was explained in the “Patients” section, for each patient P*i*, *i* = 1, …, 31, three whole-body scans were obtained: the baseline scans and the two follow-up scans. Each one of these three sequences comprises of about 400 images. The specifications of the PET images are given in Table 1.

**Table 1:** General features of PET images

Specifications of PET images	
Image size	400 × 400 pixels
Pixel size	2.03642 × 2.03642 mm^2^
Slice thickness	4 mm

It must be noted here that FDG also concentrates physiologically in several healthy organs where metabolic activity takes place. These organs are the heart, the brain, the liver, the urinary tract (due to tracer extraction) etc. Because it is not possible to systematically differentiate between non-malignant enhanced FDG activity (e.g. in inflammatory lesions) and abnormal activity in the tumorous lesions, a systematic error is introduced in the calculations of the fractal dimensions. This systematic error will be discussed further in the “Results” section.

### Data analysis

Evaluation of the PET/CT data was performed using dedicated software (Aycan Digitalsysteme, Würzburg, Germany). Visual analysis was performed by two nuclear medicine physicians evaluating the hypermetabolic areas on transaxial, coronal and sagittal images which were considered to be malignant. Patient history was studied thoroughly in order to exclude possible causes of non-specific tracer accumulation and therefore minimize false positive findings. The overall treatment outcome was determined by the dermatooncologists and included further parameters, like clinical data and radiological imaging modalities.

### Fractal and multifractal analysis

As discussed in the “Background”, melanoma is a fast evolving tumour which expands forming numerous lesions throughout the body. To quantify the spatial extension of the metastases, we use fractal analysis which takes into account only the locations of the lesions, while multifractal analysis accounts also for the size of the metastases in each affected area. The advantage of using fractal analysis to address this problem is that there exist a number of growth models/schemes which give rise to fractal structures [21–27]. Depending on the calculated fractal dimension, we can then point to the appropriate mechanisms most accounting for the spreading of the tumours.

From the many measures which are proposed for calculating fractality of the spreading, we use here the most classic one, the box-counting dimensions [2, 18, 19]. This measure has the advantage of being easy to implement and it gives directly a means of differentiating between homogeneous and hierarchical arrangement of the lesions.

To implement the box-counting method, we divide the 3D PET/CT image of the human body in cubic boxes (3D pixels-voxels) of linear size *s***.** The voxel linear size was chosen considering the pixel size and the image thickness (Table 1). According to the image specifications, the pixel size is approximately 2 mm × 2 mm and the image thickness is 4 mm. To be consistent in the three directions, we construct cubic boxes with a minimum length of *s*_min_ = 4 mm up to *s*_max_ = 200 mm.

For each value of box size *s*, we calculate the number of cubic boxes *N*(*s*) which contain tumour cells (primary or metastatic). The presence of tumour cells in a given position is recorded by the presence of the tracer. By plotting *N*(*s*) as a function of *s*, we extract the fractal dimension *d*_f_ fitting the data to the following formula:

1 $$ N(s)=C{s}^{-{d}_{\mathrm{f}}} $$

where *C* is a constant, determined also by the above fit. Alternatively, we can plot *N*(*s*) as a function of *s* in a double logarithmic scale and the fractal dimension is calculated as the slope of this curve.

In these calculations, a systematic error arises because the tracer accumulates not only in malignant regions but also in some healthy organs as well as in non-tumour-related findings (e.g. inflammatory lesions). This error tends to increase the fractal dimension towards the value 3, since the organs contribute substantial accumulations of tracer in 3D volumes.

While fractal analysis uses only the presence of the tracer in the particular lesion, multifractal analysis takes also into account the concentration of the tracer which is accounted for by the intensity *p*_*i*_ of the colour in each voxel *i*. The colour intensity is normalized so that ${\displaystyle \sum_{i=1}^N}\ {p}_i=1$, in order to get results which do not depend on the particular average of the individual subjects, but only on the scaling of their data. In this case, we use the smallest box sizes available, because the multifractal spectrum requires the finest details of the spatial structure. For the present scans, the smallest box size available is (4 mm × 4 mm × 4 mm). The generalized dimensions *D*_*q*_ (or multifractal spectrum) are calculated as follows:

2 $$ \begin{array}{c}\hfill Dq={\displaystyle \underset{s\to 0}{ \lim }}\frac{1}{q-1}\kern0.5em \frac{1}{ \ln \kern0.2em s} \ln \left({\displaystyle \sum_{i=1}^N{p}_i^q}\right),\kern0.5em q\ne 1\hfill \\ {}\hfill {D}_1={\displaystyle \underset{s\to 0}{ \lim }}\frac{1}{ \ln \kern0.2em s}{\displaystyle {\sum}_{i=1}^N{p}_i\kern0.5em  ln}\kern0.5em {p}_i\kern0.5em ,\kern0.5em q=1\hfill \end{array} $$

where *q* is the dimension index which takes real values (−∞, … , 0, … ,+∞), *s* = 4 mm, the index *i* runs over all boxes and *N* is the total number of boxes of size (4 mm × 4 mm × 4 mm) covering the body. Note that the negative values of *q* highlight the smallest values of *p*_*i*_ (rare events), while the larger positive values of *p*_*i*_ are accentuated by the positive values of *q*.

Finally, the integral difference of the multifractal spectrum, average multifractal index *D*_MF_, between studies I and II (early), II and III (late) and I and III (final) is useful, because it gives the tendency of the spectrum, averaged over all *q* values. Formula 3 is used for the calculation of *D*_MF_ between stages I and II,

3 $$ {D}_{\mathrm{MF}\left(I,II\right)}=\frac{1}{2L+1}{\displaystyle \sum_{q=-L}^L\left({D}_q\left(\mathrm{I}\right)-{D}_q\left(\mathrm{I}\mathrm{I}\right)\right)} $$

and similarly between stages II and III and between stages I and III. In 3, *L* (−*L*) is the highest (lowest) order of index *q*. When *D*_MF_ takes positive values, the patient improves, while for negative values his/her condition deteriorates.

For the computations of the fractal dimensions, the multifractal spectrum and the average multifractal index, we use in-house software adjusted to the specific needs of the PET/CT imaging data.

### The Kinetic Monte Carlo method

Previous attempts to model the growth of tumours include both continuous and discrete approaches [28]. Most of these models deal with the growth of a single lesion as a result of the increase of the number of malignant cells locally. The propagation of tumour cells causing metastases has been studied using mainly continuous diffusion models [29–31]. In the current study, we employ a stochastic microscopic approach, which allows the malignant cells to move randomly through the blood circulatory system [21–23]. To model the metastatic process of melanoma, we allow a small number of malignant cells to diffuse through the blood circulatory system and to cause occasional metastases in the adjacent healthy tissue or organs.

More specifically, earlier studies have shown that the blood circulatory system has a branching structure whose geometry is fractal [3, 6]. As recapitulated in the “Background”, the fractal dimensions recorded in the literature is of the order of 2.6–2.7. The method of Kinetic Monte Carlo (KMC) simulations for the specific application of the creation of metastatic lesions consists in constructing the blood circulatory network as a fractal medium, embedded in the 3D space representing the human body. The human body is then constructed as a 3D matrix consisting of (a) cells which represent the fractal circulatory system, (b) cells which represent tissue and organs where metastasis can take place and FDG is accumulated and (c) other unoccupied (empty) areas. In this system, a small number (*n*) of malignant cells are released to diffuse, while their motion is restricted only within the areas covered by the circulatory network, i.e. they do not enter the areas outside the circulatory system [21, 24]. The motion of the malignant cells is described as a classical random walk (as assumed in this study) or a directed random walk or an anomalous walk [25, 26]. Occasionally, with very small probability (*p*) they invade the tissue (or organs) adjacent to the circulatory system. The system is integrated for a long time and as KMC time passes, the number of tumour sites increases and they span more and more areas around the body, all of them adjacent to the circulatory system. In this way, a qualitative lesion distribution is obtained whose statistical characteristics convey attributes similar to the ones recorded from the medical images, provided the parameters of the KMC model are representative of the evolution of the disease.

To calculate the fractal dimensions of the KMC simulation data, we use the same methods and in-house software as for the medical data, i.e. we use the box-counting algorithm described in the “Fractal and multifractal analysis” section to calculate the *d*_f_ from the simulations, for direct comparison with the results from the PET/CT images.

## Results

### Typical patients

For each patient, we calculated the box-counting dimensions according to Formula 1, before medical treatment (study I), after two cycles of ipilimumab treatment (study II) and at the end of the four-cycle treatment (study III). The results for all subjects are given in Table 2. First, the results of the 31 patients are recorded, followed by the 2 healthy subjects.

**Table 2:** Analysis results for all patients

Patient no.	Age (years)	Sex (M/F)	Study	Treatment response	Fractal dimension	Average multifractal index	Matching results
P1	67	M	Early	^a^	2.487	−0.064	Typ
			Late	^a^	2.509	−0.030	
			Final	PR	2.525	−0.095	
P2	48	F	Early	PD (slow)	2.611	+0.043	Typ
			Late	MR	2.588	−0.029	
			Final	MR	2.623	+0.013	
P3	56	M	Early	^a^	2.465	−0.119	Typ
			Late	^a^	2.644	−0.015	
			Final	PR	2.643	−0.135	
P4	62	M	Early	PD	2.688	−0.108	Typ
			Late	MR	2.743	+0.003	
			Final	PD	2.726	−0.104	
P5	37	F	Early	PD (slow)	2.512	+0.181	Typ
			Late	PD (slow)	2.395	−0.308	
			Final	PD (slow)	2.602	−0.127	
P6	55	M	Early	PD	2.634	−0.024	Typ
			Late	PD	2.673	+0.174	
			Final	PD	2.511	+0.150	
P7	55	M	Early	PD	2.65	+0.077	Typ
			Late	SD	2.629	−0.024	
			Final	PD	2.624	+0.053	
P8	74	M	Early	PD (slow)	2.622	+0.01	Typ
			Late	SD	2.629	+0.11	
			Final	PD	2.529	+0.13	
P9	60	F	Early	PD	2.618	+0.016	Non-Typ+
			Late	PD	2.603	−0.14	
			Final	PD	2.655	−0.124	
P10	67	M	Early	SD	2.589	−0.065	Non-Typ
			Late	PD	2.633	−0.044	
			Final	PD	2.652	−0.11	
P11	56	M	Early	MR	2.453	−0.369	Typ
			Late	PD (slow)	2.719	+0.455	
			Final	MR	2.4	+0.085	
P12	55	M	Early	PD (slow)	2.637	+0.044	Typ
			Late	PD	2.591	+0.03	
			Final	PD	2.56	+0.075	
P13	71	M	Early	SD	2.653	−0.113	Typ
			Late	SD	2.676	+0.07	
			Final	PR	2.683	−0.043	
P14	36	M	Early	PD	2.616	+0.053	Typ
			Late	PD	2.613	+0.106	
			Final	PD	2.523	+0.159	
P15	55	M	Early	SD	2.593	−0.264	Non-Typ+
			Late	SD	2.707	+0.281	
			Final	SD	2.575	0.017	
P16	73	M	Early	SD	2.61	−0.075	Typ
			Late	PD (slow)	2.676	+0.063	
			Final	PD (slow)	2.618	−0.012	
P17	61	F	Early	PD (slow)	2.698	+0.001	Typ
			Late	PR	2.697	−0.033	
			Final	PR	2.705	−0.031	
P18	71	M	Early	PD	2.697	−0.031	Non-Typ+
			Late	PD	2.684	−0.047	
			Final	PD	2.713	−0.078	
P19	39	M	Early	PD (slow)	2.611	−0.036	Typ
			Late	SD	2.629	+0.088	
			Final	PD (slow)	2.572	+0.052	
P20	70	M	Early	PD	2.663	+0.045	Typ
			Late	PD	2.659	−0.021	
			Final	PD	2.621	+0.024	
P21	49	M	Early	MR	2.533	+0.012	Non-Typ
			Late	PD	2.518	−0.036	
			Final	PD	2.556	−0.023	
P22	66	M	Early	SD	2.572	−0.037	Typ
			Late	SD	2.561	+0.031	
			Final	SD	2.582	−0.005	
P23	75	M	Early	PR	2.68	+0.09	Non-Typ+
			Late	PD	2.609	+0.011	
			Final	PD	2.651	+0.101	
P24	67	M	Early	SD	2.687	+0.03	Non-Typ+
			Late	PD (slow)	2.67	−0.071	
			Final	PD (slow)	2.741	−0.04	
P25	65	F	Early	MR	2.48	−0.319	Typ
			Late	MR	2.699	+0.151	
			Final	MR	2.614	−0.168	
P26	73	F	Early	PR	2.65	−0.081	Non-Typ
			Late	PD	2.708	−0.07	
			Final	PD	2.714	−0.151	
P27	62	M	Early	PD	2.602	+0.021	Non-Typ+
			Late	PR	2.599	+0.047	
			Final	PR	2.576	+0.069	
P28	54	F	Early	PR	2.699	+0.129	Typ
			Late	PR	2.655	−0.187	
			Final	PR	2.716	−0.058	
P29	61	F	Early	PR	2.622	−0.019	Non-Typ
			Late	PR	2.632	−0.032	
			Final	PR	2.651	−0.052	
P30	53	M	Early	SD	2.544	+0.051	Typ
			Late	SD	2.534	−0.203	
			Final	SD	2.676	−0.152	
P31	78	F	Early	SD	2.588	+0.046	Non-Typ+
			Late	SD	2.554	−0.165	
			Final	SD	2.633	−0.118	
Healthy subject 1	69	F	–	–	2.69	–	–
Healthy subject 2	73	M	–	–	2.723	–	–

Early stage: study I → study II; late stage: study II → study III; final stage: study I → study III

Typ: correspondence between analysis results and treatment outcome data

Non-Typ: non-typical patient (unknown reasons)

Non-Typ+: non-typical patient due to unspecific findings

*PR* partial remission, *SD* stable disease, *PD* progressive disease, *MR* mixed response

^a^No treatment outcome data

The general analysis and comparison between the fractal dimensions before and after treatment shows that the tracer has the tendency to spread in higher dimensionality in healthy subjects and also when the treatment is successful. Typical healthy subjects demonstrate fractal dimensions (in physiological distribution of tracer) around 2.7 (see healthy subjects 1 and 2, at the end of Table 2). Since the tracer is not accumulated by malignant lesions in healthy subjects, it tends to spread almost homogeneously throughout the 3D extension of the body, or concentrates in the 3D volume covered by organs in which biochemical activity takes place. In these cases, the spreading is expected to show fractal dimensions almost equal (very close) to 3. The results from the PET/CT images of healthy subjects show smaller fractal dimensions because of the different tracer distribution in the organs, including some organs like the liver and brain that physiologically accumulate FDG. Both these factors lower, in the statistical sense, the fractal dimensions in healthy subjects below 3.

When lesions are spreading through the body, the tracer starts to accumulate in areas where the metastases are active. If we make the assumption that the spreading takes place through the blood circulatory network and that the metastases are engendered around it, we expect that the spreading at the beginning will start having small extension (thus small fractal dimensions) while at later stages the lesions’ spatial extension will increase, approaching at most the spatial extension (and thus the fractal dimensions) of the circulatory network. (Corrections to these calculated values must be taken into account due to the tracer accumulation by healthy organs). These propositions are in agreement with the findings, using the patients’ treatment outcome as reference, that (a) when the treatment with ipilimumab leads to improvement of therapy outcome the fractal dimension increases as the patient improves, (b) when the patient’s condition is stable, *d*_f_ does not change drastically and (c) when the patient’s condition deteriorates *d*_f_ takes smaller values because the tracer concentrates further in the active lesions rather than in normal organs.

**Fig. 1:**
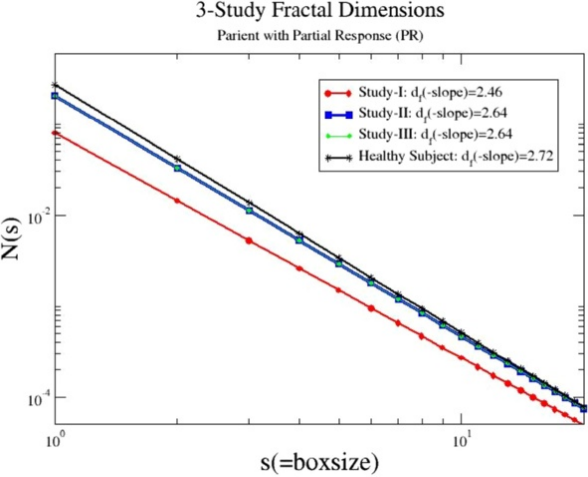
The number of boxes *N*(*s*) as a function of the box size *s* for patient P3. His/her condition improves after medical treatment with ipilimumab. The *solid lines* represent the linear regressions to the data

A representative plot for patient P3 is shown in Fig. 1. With the red line, we represent the analysis of study I, when the metastatic melanoma was diagnosed, with the blue line is the analysis after two cycles of ipilimumab treatment (study II) and with the green line the analysis after four cycles of treatment (study III). As indicated by the treatment outcome, the patient at the baseline study I showed low fractal dimensions *d*_f_(I) = 2.46 (metastatic invasion). After two cycles, we have an improvement of the patient’s condition and *d*_f_(II) increased to 2.64, and after four cycles, *d*_f_(III) did not change substantially, showing a stable phase of the disease. In the same figure, the line corresponding to the analysis of the Healthy Subject (2) is plotted, for comparison.

**Fig. 2:**
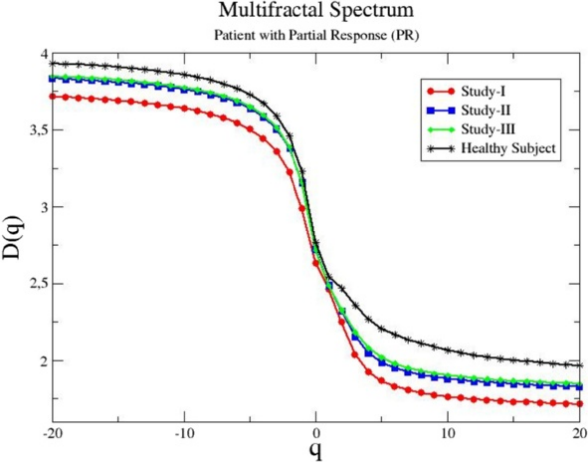
The generalized dimensions *D*_*q*_ as a function of the index *q* for patient P3. The patient’s condition improves after medical treatment with ipilimumab

The results of the multifractal spectrum verify further the fractal analysis. For the same patient, the generalized dimensions are presented in Fig. 2. Again, the red line which corresponds to the initial diagnosis of the disease (study I) is lower than all other lines. The blue line which records the analysis after two cycles of treatment (study II) and the green line, after four cycles (study III), are at the same level, higher than the baseline spectrum. This indicates improvement in the patient conditions and elimination of malignant lesions. Again, the black line which denotes the multifractal spectrum of the healthy subject is plotted in Fig. 2, for comparison.

**Fig. 3:**
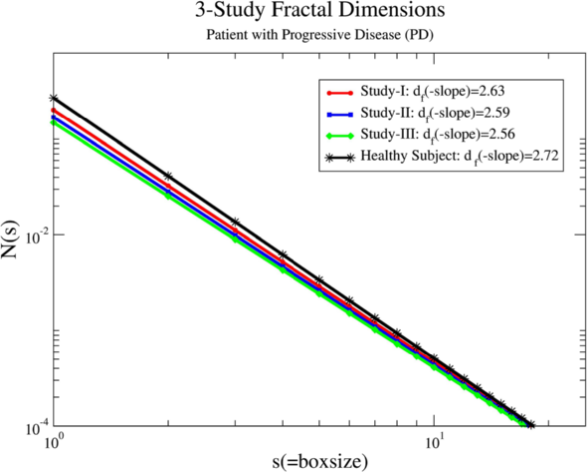
The number of boxes *N*(*s*) as a function of the box size *s* for patient P12. His/her condition deteriorates during medical treatment with ipilimumab. The *solid lines* represent the linear regressions to the data

**Fig. 4:**
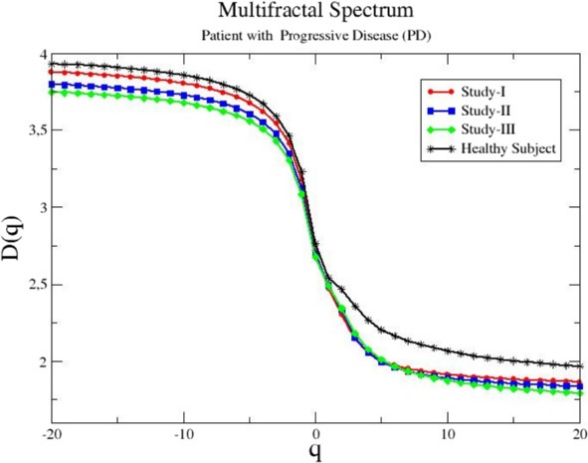
The generalized dimensions *D*_*q*_ as a function of the index *q* for patient P12. The patient’s condition deteriorates during medical treatment with ipilimumab

Another representative plot from patient P12, whose condition according to the treatment outcomes deteriorates during therapy, is shown in Fig. 3. In this case, the *d*_f_ value drops successively among the different studies. On study I, the fractal dimension showed the highest value *d*_f_(I) = 2.63. As the disease progressed, a decrease in *d*_f_ values is shown with *d*_f_(II) = 2.59 and *d*_f_(III) = 2.56. The above confirms the hypothesis that in the presence of developing malignant lesions the tracer tends to accumulate in them, showing hierarchical expansion of low dimensionality. Figure 4 presents the multifractal spectrum for the same patient. The curve shapes corroborate the results of the fractal analysis showing a similar decreasing tendency.

Comparatively, all the results for all patients are recorded on Table 2. In the first column, the patient index is given, as P1, P2, …, P31. In the second and third columns, the patient’s age and sex are recorded. In the fourth column, the three therapy stages are set, while in the fifth column the treatment outcomes are reported based on clinically used criteria for therapy response evaluation. We characterize the progression of the disease between studies I and II (Early), studies II and III (Late) and studies I and III (Final), according to treatment outcome as defined by the referring oncologists. The patient’s condition is characterized by the oncologists as partial remission (PR) if the patient improves after treatment, as stable disease (SD) if the patient’s condition is stable, as progressive disease (PD) if the patient deteriorates and as mixed response (MR) in case of good response in some lesions and non-response in others. We used the term of MR to describe the non-uniform response to treatment, which is often the case due to tumour heterogeneity. The sixth column records the fractal dimension characteristic of the particular study. In the seventh column, the early, late and final average multifractal indices are reported, using Formula 3. In the last column, the patient is characterized as standard/typical (Typ) if he/she only suffers from metastatic melanoma and the comparison of the analysis results and the treatment outcome results follow the typical pattern as explained in the “Typical patients” section.

The patient is characterized as non-typical + (Non-Typ+) if he/she has a non-tumour-related finding or as non-typical (Non-Typ) if the results of the fractal analysis were not consistent with the treatment outcome data for unknown reasons. Patients characterized as Non-Typ**+** are individually discussed in the next section.

### Unspecific tracer uptake not related to melanoma disease

In this section, we present two cases of non-typical patients, both with medical findings not directly related to the melanoma disease. In both cases, there is an uptake of FDG in non-tumorous areas and thus the results of the fractal/multifractal analysis are incompatible with the treatment outcome data.

#### The case of colitis

**Fig. 5:**
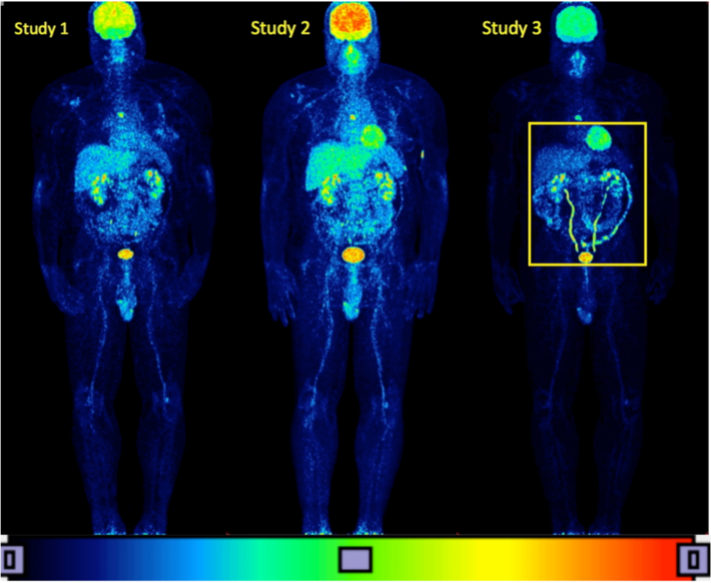
PET/CT images of patient P15 suffering from colitis. The patient presents high uptake of the FDG biomarker not only due to the melanoma metastases but also due to the presence of colitis, not related to the melanoma

Patient P15 who suffers from metastatic melanoma presents one metastatic lesion in the sixth thoracic vertebra, which is clearly delineated in the maximum intensity projection images in both follow-up studies (see Fig. 5). The same patient suffers also from fast evolving colitis, which is a side-effect of ipilimumab therapy and this is evident from the high uptake of FDG in the colon as shown on study III. Furthermore, there is tracer retention in both ureters in the third study, which is also a finding not related to the melanoma disease. Thus the distribution of FDG is divided between healthy organs, metastatic lesions and the colon area. This introduces a large error in the calculations, and as a result, the fractal dimension increases in the study II and decreases in the study III, while the patient’s condition remains practically steady.

#### The case of unspecific FDG uptake (thrombophlebitis)

**Fig. 6:**
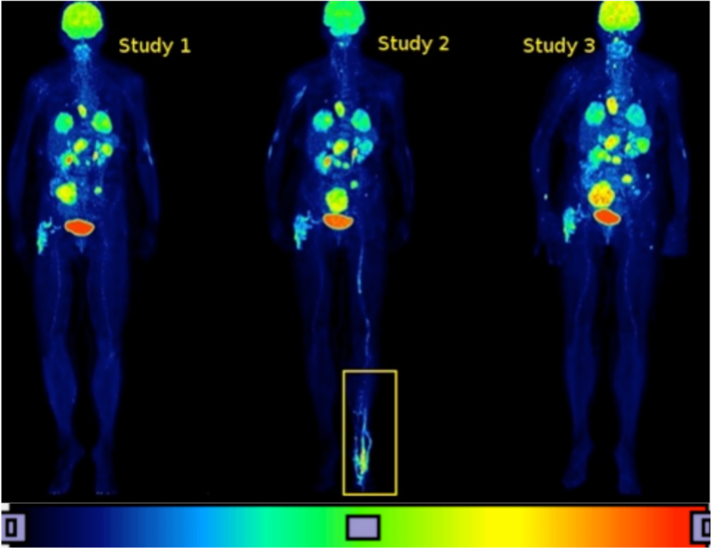
PET/CT images of patient P31 at the three stages. The patient presents high unusual uptake of the FDG biomarker in his/her left leg at the second stage of treatment, not related to the melanoma

Patient P31 in the stage of treatment (study II) presents an increase in FDG uptake in the left leg which disappears in the study III. This uptake is not related to fast progressing melanoma but to an inflammatory vessel disease (see Fig. 6). Due to this higher dispersion of the FDG biomarker, the fractal dimension decreases in study II, as if the patient was deteriorating (developing further lesions) while the treatment outcome data indicate that the patient’s condition is stable. Similar, hypermetabolic lesions not directly related to melanoma (unspecific findings) altering the results of the fractal/multifractal analysis are met in 7 out of 31 patients. These patients are marked as Non-Typ+ in Table 2.

The results of this section demonstrate that the fractal/multifractal analysis predicts correctly the evolution of metastatic melanoma in 83.3 % (20 in 24) of the cases, giving results compatible with the treatment outcome data. In 7 cases, combined effects of melanoma and other non-specific findings (e.g. inflammatory lesions) give results non-compatible with treatment outcome data. We stress again that the fractal/multifractal analysis of the metastatic melanoma spreading must be always complemented with medical assessment in order to exclude regions with false positive FDG uptake not related to the disease.

### Kinetic Monte Carlo simulations

For this study, the blood circulatory network was constructed as a deterministic fractal embedded in 3D space representing the human body. The system (body) size was *L* × *L* × *L* = 81 × 81 × 81 sites (3D representation), and the deterministic fractal network corresponding to the circulatory system was constructed iteratively within this 3D space. The fractal network spans the body and its fractal dimension *d*_f_ was chosen *d*_f_^c^ = ln(18)/ln(3) = 2.69 to be similar to the value reported in the literature. Once the positions of the sites belonging to the circulatory system were assigned, a small number (*n* = 10) of malignant cells were released to diffuse only within the areas covered by the circulatory network. Occasionally, with very small probability (*p*), they infect the tissue (or organs) adjacent to the circulatory system. As time passes, the number of tumour lesions increases and they span more and more areas around the body, all of them adjacent to the circulatory system.

**Fig. 7:**
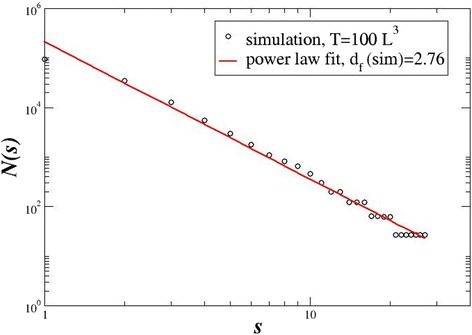
The number of boxes containing metastases as a function of the box size *s*. The linear system size is *L* = 81, the probability of infection is *p* = 0.001, the infecting seeds are *n* = 10 and the time of the simulation is *T* = 100 × *L* ^3^

To find the characteristics of the spatial distribution of the metastatic lesions produced by the KMC model, we use the same algorithm (box-counting method) as in the calculations of the fractal dimensions of the PET/CT images, i.e. we divide the 81 × 81 × 81 sites in cubic boxes of size *s* = 1, 2, …, 20, and we calculate the number of boxes *N*(*s*) which contain at least one infected site. In a double logarithmic scale, the tangent of the number of boxes *N*(*s*) versus the box size *s* expresses the fractal dimensions *d*_f_(sim)*.* The results of a typical simulation are shown in Fig. 7. In this simulation, each malignant cell diffuses for time *T* = 100 × *L*^3^ where *L* is the linear size of the body (in voxels). At every elementary time step, they diffuse randomly into nearest neighbouring sites (provided that they belong to the circulatory system), and with probability *p* = 0.001, they cause metastases in the nearby tissues or organs.

**Fig. 8:**
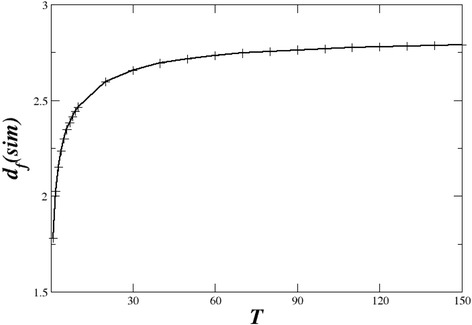
The temporal evolution of *d*_f_(sim)

The number of metastatic regions at this time shows a typical power law with exponent *d*_f_(sim) = 2.76, close to the one of the circulatory system. In earlier times, the metastases distribution is more sparse and the evolution of the fractal dimension with time is shown on Fig. 8. This figure shows that as time increases the fractal dimension of the metastatic lesions approaches closely the one of the circulatory system. In fact, it tends to be a little higher. This is explained because the lesions are located all around the blood network covering a larger area than the network itself. Consequently, the corresponding fractal dimension tends to be slightly higher, in the limit of large times. We believe that the recorded PET/CT scans represent earlier, transient times before the malignant cells have the time to invade all the maximum body “volume” around the circulatory system.

**Fig. 9:**
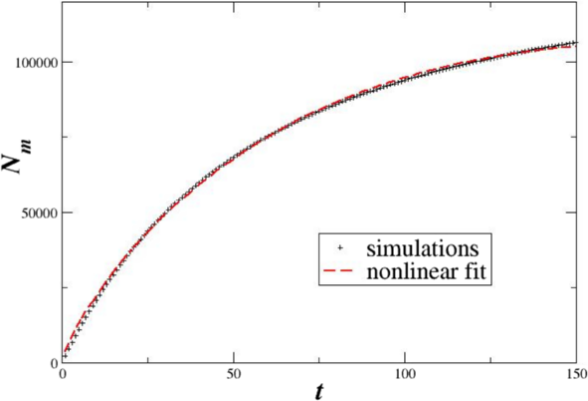
Evolution of the number of metastatic sites with time. The *crosses* represent the simulation results and the *red dashed line* the nonlinear curve fit

Fractal laws have been also demonstrated in the time activity curve of FDG in different tumours [32]. A similar tendency is demonstrated by the number of metastatic sites *N*_m_ as a function of time shown in Fig. 9. *N*_m_ increases at the beginning while after the transient it approaches a constant value, since most regions around the circulatory system have been infected and secondary infection of the same site is not allowed in the simulations. In the same figure, the simulation results are approximated using a nonlinear curve fit of the type [24]:

4 $$ {N}_{\mathrm{m}}(t)=A{t}^{\alpha } \exp \left(-\beta t\right) $$

This formula takes into account the power law (fractal) nature of the problem, expressed by the exponent *α* and the levelling up of the curve which is attained by the exponential decay term exp(−*βt*). The nonlinear curve fit gives parameter values *α* = 0.808 and *β* **=** −0.0045, with correlation coefficient 0.999697. The number of infected/malignant sites *N*_m_ cannot be directly correlated with the number of observed metastatic lesions in the PET/CT images. Each metastatic site may expand increasing the size of the lesion, but this feature has not been considered in the present version of the KMC model.

The minimal KMC model is a mechanistic scheme for the spreading of metastases in the human body. It accounts for the fractal spreading as calculated from the PET/CT images and has a similar fractal dimension. This model needs to be further explored in order to account for more precise metastasis characteristics. Extensions and improvements to the KMC model are discussed in the “Open problems and limitations” section.

## Discussion

In this section, we first present the main results of the PET/CT fractal analysis in the “Fractal and multifractal properties of the PET/CT images” section followed by the results produced by the KMC model in the “Kinetic Monte Carlo simulations and results” section, for comparison. The limitations of these approaches and algorithms are discussed in the “Open problems and limitations” section.

### Fractal and multifractal properties of the PET/CT images

We have used the methods of fractal and multifractal analysis to quantify the spatial extension of the malignant lesions in patients with metastatic melanoma, before and after treatment with ipilimumab. We analysed the PET/CT data from 31 patients prior, early in the course of treatment and immediately after the end of the treatment. The fractal dimensions show low, decreasing values in patients with progressive disease, demonstrating the tendency of the tracer to concentrate in the sub-region of the body where metabolic activity takes place. However, in cases of successful medical treatment, the lesions cease to attract the tracer and the fractal dimensions increase, showing the tendency of the tracer to diffuse in all parts of the body. In 20 out of 24 cases (83.3 %), the results of the fractal and multifractal analysis were consistent with the treatment outcome data. For the remaining seven patients, non-tumour-related medical conditions led to abnormal tracer uptake, which gave false quantitative results. Two of these cases were demonstrated in detail in the “Unspecific tracer uptake not related to melanoma disease” section as common atypical cases.

### Kinetic Monte Carlo simulations and results

To understand the quantitative results, we have devised the stochastic KMC model, based on the random drift of malignant cells through the blood circulatory system, which colonize randomly the surrounding tissue. The main results of the KMC model is that as the malignant cells diffuse through the blood network colonizing at first contact (with given, small probability), the colonies at the beginning of the process seem to be scattered randomly in the body. As time evolves, and more and more metastases occur, the metastatic topology will develop following the shape of the accommodating blood circulatory (or other hosting) network. This is clearly shown in the time evolution of the fractal dimensions, which as time evolves approach the limiting set with *d*_f_ = 2.7, characteristic of biological networks. The spatial distributions of metastases produced by the KMC model are statistically similar to the ones observed in melanoma patients.

The number of malignant sites in the KMC model is shown to follow a mixed mathematical increasing expression containing both exponential and power law parts, characteristic of fractal growth phenomena [27]. This mathematical formula can be used as predictor for the extension of the “volume” of the metastatic lesions with time, provided that more frequent scans of the patients are available in order to have enough data to accurately fix the values of the constants *A*, *α* and *β* in Formula 4.

### Open problems and limitations

One limitation of the present study is the selectivity of the algorithm used for the calculation of the fractal and multifractal dimensions. An important improvement to the algorithm would be the ability to exclude healthy organs where the tracer physiologically accumulates, as well as areas which show an unspecific non-tumour-related FDG uptake. This became evident during the data evaluation and after the comparison between the results of the fractal analysis and the treatment outcome data. It is possible to overcome this problem either with the development of new, smart algorithms able to mask areas with non-tumour-related tracer uptake, or by using a specific tracer, which does not show accumulation in inflammatory areas. Other nonlinear measures such as the spatial correlations, higher order moments of the spatial distribution and clustering coefficients can be used to characterize the metastatic potential of the tumours and can improve the predictions of therapy outcome. These measures can be easily incorporated in the existing algorithms.

The results of the changes on *d*_f_ and *D*_*q*_ presented in Table 2 are suggestive of the evolution of melanoma, but it would be useful to establish the ranges of these measures in the healthy subjects and patients with different tumorous stages. Because melanoma is a disease which evolves fast (and so does *d*_f_ and *D*_*q*_), it would be useful to clinically classify various stages of melanoma and specify the ranges of *d*_f_ and *D*_*q*_ associated with each stage, including the healthy stage. Even with a limited number of subjects, the use of random resampling statistical methods allows to safely determine the *d*_f_ and *D*_*q*_ confidence intervals, provided that the subjects are properly classified at the various stages of the disease [33–36].

A direct improvement to the KMC model would be to choose a spatial extension similar to the one used in PET/CT (e.g. 400 × 400 × 400 depending on the patient height) rather than the simple cubic (81 × 81 × 81) used here. Instead of using a deterministic fractal, we could mimic the circulatory network itself, drawing from medical images. We can also vary the number of malignant cells which circulate in the blood vessels and make their motion more realistic, rather than classical diffusion. It is also possible to arrange the malignant cells to start from a single tumorous area (e.g. the primary tumour) rather than spreading them randomly in the circulatory network. These additions to the KMC model could lead to even closer predictions of the spatial distribution of the lesions in metastatic melanoma.

## Conclusions

In this study we present a new method based on the calculations of fractal and multifractal dimensions of PET/CT images with FDG tracer for monitoring of the therapeutic effect of ipilimumab in patients with metastatic melanoma. The results show enough evidence that the fractal and multifractal analyses have the potential to serve as biomarkers, for therapy monitoring and for following the evolution of metastases in the body. This is a robust, operator-independent method which is based on static images, can be easily implemented and demonstrated a correct classification rate of 83.3 % in this study. It can be used as an additional quantitative parameter for the assessment of the therapeutic outcome in clinical routine in particular within a multiparameter evaluation approach [13]. Additional studies over a larger number of patients are needed to verify the impact of this method, as well as refinement of the developed algorithms to improve their predictive value.
